# Valsartan Attenuated Homocysteine-Induced Impaired Autophagy and ER Stress in Human Umbilical Vein Endothelial Cells

**DOI:** 10.1155/2023/8817431

**Published:** 2023-12-13

**Authors:** Xinyan Wu, Ani Wang, Long Xu, Meng Li, Qingxian Zhai, Weidong Wang, Chunling Li, Lizi Jin

**Affiliations:** ^1^Department of Cardiology, The 5th Affiliated Hospital, Sun Yat-sen University, Zhuhai 519000, China; ^2^Department of Pathophysiology, Zhongshan School of Medicine, Sun Yat-sen University, Guangzhou 510080, China; ^3^Department of Physiology, Zhongshan School of Medicine, Sun Yat-sen University, Guangzhou 510080, China

## Abstract

Hyperhomocysteinemia is a risk factor for various cardiovascular diseases. However, the mechanism underlying homocysteine- (Hcy-) induced vascular injury remains unclear. The purpose of the present study was to examine a potential mechanism by which Hcy induced injury in human umbilical vascular endothelial cells (HUVEC). The protein abundance of autophagy-related markers was markedly decreased after Hcy treatment, which was associated with endoplasmic reticulum (ER) stress and apoptosis in HUVECs. Protein expression level of angiotensin II type 1 receptor (AT1 receptor) was dramatically increased in response to Hcy. Valsartan, an AT1 receptor blocker, improved autophagy and prevented ER stress and apoptosis in HUVECs treated with Hcy. Consistent with this, silence of AT1 receptor with siRNA decreased the protein abundance of ER stress markers, prevented apoptosis, and promoted autophagy in HUVECs. Inhibition or knockdown of AT1 receptor was shown to be associated with suppression of p-GSK3*β*/GSK3*β*-p-mTOR/mTOR signaling pathway. Additionally, inhibition of autophagy by 3-MA aggravated Hcy-induced apoptosis, while amelioration of ER stress by 4-PBA prevented Hcy-induced injury in HUVECs. Hcy-induced HUVEC injury was likely attributed to AT1 receptor activation, leading to impaired autophagy, ER stress, and apoptosis.

## 1. Introduction

Increased serum level of homocysteine (Hcy) is a risk factor for cardiovascular disease and is specifically linked to various diseases of the vasculature such as atherosclerosis and stroke. Hcy exerts its adverse effects by disturbing endothelial function, which is a key initial event in the setting of atherosclerosis [1]. The relationship between Hcy metabolism and endothelial dysfunction remains an important and exciting field of research. Several molecular mechanisms mediating cellular dysfunction and injury caused by Hcy in human umbilical vascular endothelial cell (HUVEC) lines have been investigated, including autophagy [2], increased oxidation in mitochondria [3], endoplasmic reticulum (ER) stress, and apoptosis [4].

Autophagy is an evolutionarily conserved subcellular process which is involved in the degradation of proteins and damaged organelles in lysosomal compartments [5]. Besides its role in the regulation of endothelial survival, autophagy is also involved in many other endothelial functions such as NO production, angiogenesis, and thrombosis [6, 7]. Defective endothelial autophagy is associated with an inflammatory and apoptotic phenotype.

When the cells are unable to properly fold or fail to posttranslationally modify secretory and transmembrane proteins in the ER, misfolded proteins will be built up in this organelle, causing ER stress. Persistent ER stress causes cell death and has been suggested to play a role in the development of endothelial dysfunction, while inhibition of the ER stress response pathways was shown to provide cardiovascular protection [8]. Although ER stress and autophagy can function independently, they share a number of common features, such as protecting cells by relieving stress and inducing cell death under extreme conditions. Alteration of one of these two cellular events can influence the other. Autophagy and ER stress keep balance in order to maintain cellular homeostasis and survival. Blocking autophagy is shown to enhance ER stress-induced cell death [9]. Defective autophagy caused ER stress and led to cell death [10], while unsolved ER stress can evoke autophagy to maintain homeostasis [11].

Many factors may induce autophagy and ER stress, such as overloaded calcium, palmitic acid [10, 12], and stimulation of angiotensin II type 1 receptor (AT1 receptor) [13]. Local activation of renin-angiotensin system plays an important role in endothelial injury. Activation of AT1 receptor is involved in vascular and cardiac hypertrophy, cardiac remodeling, endothelial dysfunction [14], and neointima formation [15], leading to atherothrombosis [14]. Angiotensin II (Ang II) was shown to induce autophagy and apoptosis in HUVECs [16] and ER stress in cardiomyocytes [17]. Hcy was also shown to directly interact and activate the AT1 receptor, aggravating vascular injury [18].

In the current study, we demonstrated that Hcy-induced HUVEC injuries likely occur through activating the AT1 receptor and downstream GSK3*β*/mTORC pathway, leading to a disbalance between autophagy and ER stress, which caused apoptosis in HUVECs.

## 2. Materials and Methods

### 2.1. Cell Culture and Treatment

Human umbilical vascular endothelial cell (HUVEC) lines (CRL-1730) were obtained from the American Type Culture Collection (Manassas, VA). Cells were cultured in a humidified incubator under 5% CO2 at 37°C using Dulbecco's modified Eagle's medium (DMEM)-F12 media (Corning) supplemented with 10% fetal bovine serum (FBS, Quacell Biotechnology).

The HUVECs were seeded on 6-well plates (Thermo Fisher Scientific) at a density of 2 × 10^5^ cells/well for 24 hours and were serum-starved for 12 hours. The medium was changed to fresh serum-free DMEM/F12 before treatment. To examine the effects of AT1 receptor blockade, HUVECs were pretreated with either 1 *μ*M valsartan (Sigma, USA) (*protocol 1*) or 1 mM Hcy (Sigma, USA) (*protocol 2*) for 30 min and then treated with Hcy or valsartan for 24 h, respectively. To determine whether PKC-GSK3*β*-mTOR pathway is involved in Hcy-induced HUVEC injury, HUVECs were pretreated with 1 *μ*M PKC inhibitor RO318820 (Tocris, UK) or 1 *μ*M GSK3*β* inhibitor TDZD-8 (MCE, China) for 30 min and then treated with 1 mM Hcy for 24 h (protocol 3).

To determine the effects of autophagy inhibition or ER stress inhibition, HUVECs were pretreated with 10 *μ*M 3-methyladenine (3-MA, MCE, China) or 1 mM 4-phenylbutyric acid (4-PBA, MCE, China) for 30 min and then treated with 1 mM Hcy for 24 h (protocol 4).

### 2.2. siRNA Transfection

Cells were transfected at 60% confluence in DMEM/F12 without FBS using Lipofectamine 3000 (Invitrogen) according to the manufacturer's instructions. The final AT1 receptor siRNA (RIBOBIO, Guangzhou, China) concentration was 100 pM.

### 2.3. Annexin V-FITC/PI Apoptosis Flow Cytometry

The extent of programmed cell death was detected by flow cytometry using Annexin V-FITC/PI apoptosis detection kit (Wanleibio, Shenyang, China). Briefly, HUVECs were pretreated with 1 *μ*M valsartan for 30 min, then treated with 1 mM Hcy for 24 h, and harvested and washed twice with PBS. The cells were centrifuged at 1500 rpm/min for 5 min and stained with Annexin V-FITC and PI in the dark, followed by flow cytometry.

### 2.4. Terminal Deoxynucleotidyl Transferase-Mediated Nick-End Labelling (TUNEL) Assay

DNA fragmentation of HUVECs was measured by TUNEL staining assay. Briefly, HUVECs were cultured on coverslips in a 24-well plate. After the indicated treatments, the cells were fixed with 4% paraformaldehyde and permeabilized with 0.3% Triton X-100. After washing with PBS, cells were incubated with TUNEL reaction mixture at 37°C in the dark for 1 h and stained by DAPI. Cells were then examined under a confocal laser scanning microscope (Leica Microsystems, Wetzlar, Germany).

### 2.5. Immunofluorescence

The HUVECs cultured on coverslips (20 mm) from different groups were rinsed with PBS (Corning), fixed with 4% paraformaldehyde, and permeabilized with 0.5% Triton X-100 for 15 mins at room temperature. After being blocked with 10% goat serum in TBST at room temperature for 1 h and incubated with primary antibodies against LC3B antibody (1 : 200, sc-376404, Santa Cruz) overnight at 4°C, the cells were washed 3 times and incubated with secondary antibodies conjugated with Alexa Fluor 555 (donkey anti-rabbit, catalog 1189904, Thermo Fisher Scientific) for 1 h at room temperature. Nuclei were stained with DAPI (Thermo Fisher Scientific). The samples were imaged using a fluorescence microscope (Leica DMi8).

### 2.6. Cell Viability Assays

The HUVECs (5 × 10^3^ cells/well) were seeded into 96-well plates. To analyze the role of AT1 receptor in Hcy-induced cell injury, the cells were treated with 1 *μ*M valsartan or not for 24 h during Hcy treatment. For the detection of the role of AT1 knockdown in Hcy-induced cell injury, the cells were transfected with siRNA-AT1 as previously described with or without treated with Hcy for 24 h, and the cell viability was determined by using the cell counting kit-8 cell viability kit according to the manufacturer's instructions (Solarbio, Beijing, China). The absorbance at 450 nm was measured using a microplate reader (BIOTECK, USA). Relative survival rates in the presence of valsartan or siRNA-AT1 were normalized to the untreated controls after background subtraction.

### 2.7. Immunoblotting

HUVECs were lysed in protein lysis buffer for 15 minutes on ice before protein was extracted. Immunoblotting was performed with primary antibodies against LC3B (1 : 3000, ab192890, Abcam), p62 (1 : 1000, ab109012, Abcam), Beclin1 (1 : 1000, #4122, Cell Signaling Technology), AT1 receptor (1 : 1000, 25343-1-AP, Proteintech), Bax (1 : 1000, 50599-2-Ig, Proteintech), Bcl2 (1 : 1000, 68103-1-Ig, Proteintech), cleaved-caspase 3 (1 : 1000, #9664, Cell Signaling Technology), BiP (1 : 1000, #63411, Cell Signaling Technology), IRE1*α* (1 : 1000, #3294, Cell Signaling Technology), XBP1 (1 : 1000, # 12782, Cell Signaling Technology), ATF4 (1 : 1000, # 11815, Cell Signaling Technology), CHOP (1 : 1000, # 2895, Cell Signaling Technology), p-GSK3*β* (1 : 1000, #9323, Cell Signaling Technology), GSK3*β* (1 : 1000, #12456, Cell Signaling Technology), p-mTOR (1 : 1000, AP0115, ABclonal), or mTOR (1 : 1000, A2445, ABclonal), respectively.

### 2.8. Statistical Analysis

Data are presented as the means ± SEM. Data were analyzed by one-way ANOVA and the Student-Newman-Keuls tests for multiple comparisons. Statistical significance was accepted at the *P* < 0.05 level.

## 3. Results

### 3.1. Hcy Treatment Induced an Impaired Autophagy and ER Stress and Activated AT1 Receptor in HUVECs

**Figure 1:**
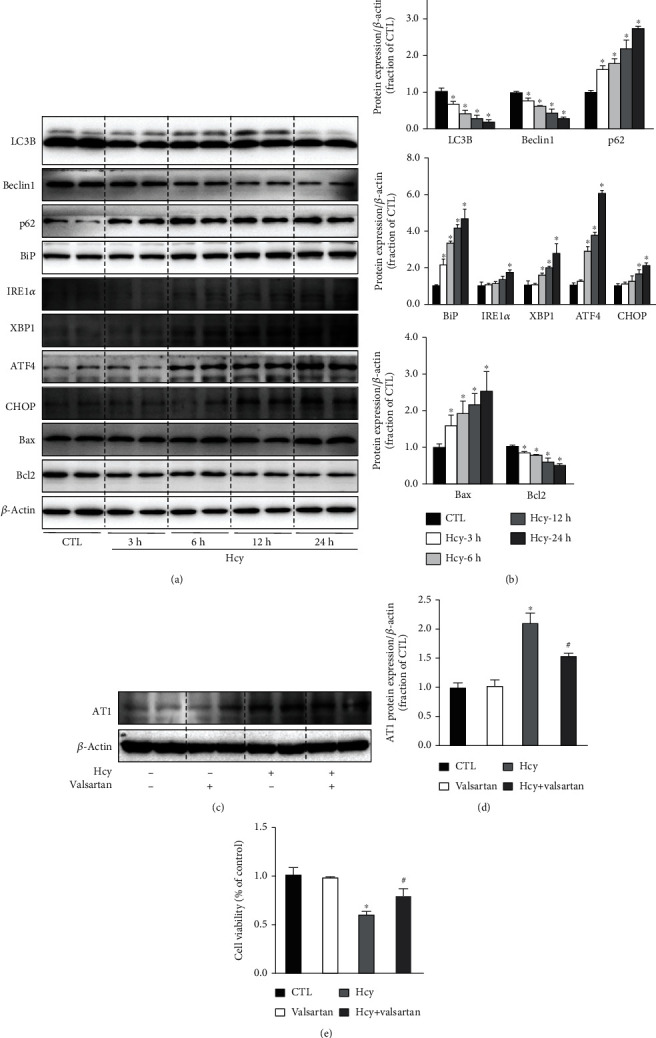
Hcy induced impaired autophagy, ER stress, and RAS in HUVECs. (a, b) Representative immunoblots and corresponding densitometry analysis of autophagy, ER stress markers, and apoptosis protein abundance in the HUVECs of CTL and Hcy treatment at 3 h, 6 h, 12 h, and 24 h. (c, d) Representative immunoblots and corresponding densitometry analysis of AT1 protein in Hcy-treated HUVECs with or without valsartan. (e) Cell viability was assessed by CCK8 assay in Hcy-treat HUVECs with AT1 receptor blocker valsartan. Data are shown as mean ± SEM; ^∗^*P* < 0.05 compared with the CTL group; ^#^*P* < 0.05 compared with the Hcy group.

To investigate the mechanisms by which Hcy-induced injury of cultured HUVECs, the expression levels of autophagy, ER stress, and apoptosis-related proteins evoked by Hcy were examined. The protein abundance of LC3B and Beclin1 was markedly downregulated since the 3 h after Hcy (1 mM) treatment and progressively decreased until 24 h, whereas p62 expression levels were markedly and consistently increased (Figures 1(a) and 1(b)). The protein expression of several ER stress-specific markers, BiP, XBP1, and ATF4, started to increase at the 6 h after Hcy treatment and dramatically increased at 24 h, while the protein expression of IRE1*α* and CHOP started to increase at the 12 h to 24 h after Hcy treatment. Apoptosis marker Bax protein level was increased at 3 h after Hcy treatment and gradually increased at 24 h. The protein abundance of Bcl2 decreased at 3 h after Hcy treatment and persistently decreased to 24 h (Figures 1(a) and 1(b)). These data suggested that Hcy induced impaired autophagy in association with ER stress, causing apoptosis in HUVECs.

Activation of AT1 receptor has been reported to be associated with HUVEC injury [19]. Western blotting revealed a marked increase in AT1 receptor abundance in HUVECs treated with Hcy, indicating an involvement of AT1 receptor in Hcy-induced cell injury. Interestingly, valsartan, an AT1 receptor blocker, moderately decreased AT1 receptor protein expression in HUVECs treated with Hcy (Figures 1(c) and 1(d)). Cell viability assay showed that inhibition of AT1 receptor by valsartan did not affect HUVEC survival, but it prevented cell injury induced by Hcy (Figure 1(e)).

### 3.2. Valsartan Prevented Hcy-Induced Impaired Autophagy and ER Stress in HUVECs

**Figure 2:**
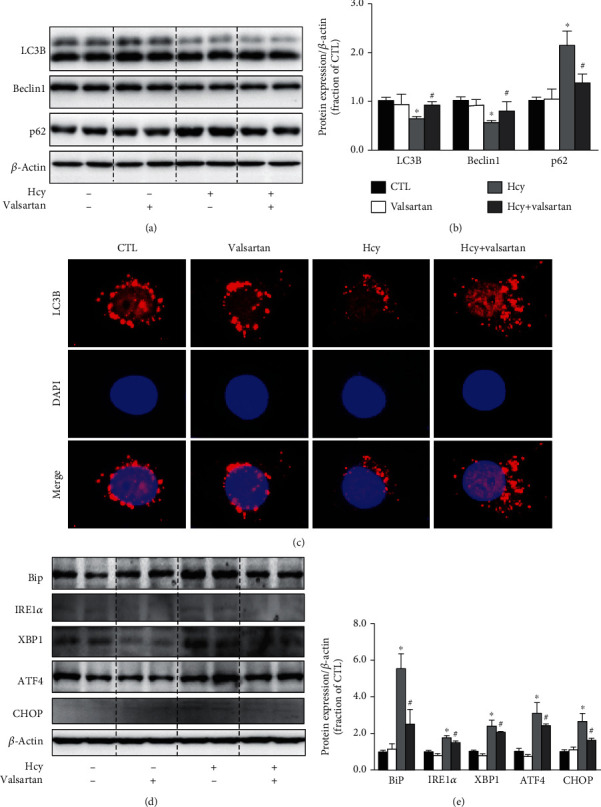
Valsartan prevented Hcy-induced impaired autophagy and ERs in HUVECs. (a, b) Representative immunoblots and corresponding densitometry analysis of LC3B, Beclin1, and p62 protein in Hcy-treated HUVECs pretreated with or without valsartan. (c) Immunofluorescence of LC3B in Hcy-treated HUVECs pretreated with valsartan. Scale bars, 5 *μ*m. (d, e) Representative immunoblots and corresponding densitometry analysis of BiP, IRE1*α*, XBP1, ATF4, and CHOP protein in Hcy-treated HUVECs with or without valsartan. Data are shown as mean ± SEM; ^∗^*P* < 0.05 compared with the CTL group; ^#^*P* < 0.05 compared with the Hcy group.

With the treatment of valsartan, an angiotensin II AT1 receptor blocker, the decreased abundance of LC3B and Beclin1 proteins induced by Hcy was markedly prevented, and the increased abundance of p62 protein was also suppressed in Hcy-treated HUVECs (Figures 2(a) and 2(b)). Immunofluorescence microscopy demonstrated that the number of LC3B puncta was less than that in the control group after Hcy treatment. Pretreatment with valsartan caused an increased formation of LC3B puncta in the Hcy group (Figure 2(c)). Valsartan also prevented Hcy-induced increased protein expression of BiP, IRE1*α*, XBP1, ATF4, and CHOP in HUVECs (Figures 2(d) and 2(e)) (protocol 1). These results indicated that AT1 receptor blockade improved impaired autophagy and ER stress induced by Hcy.

### 3.3. Valsartan Prevented Hcy-Induced Apoptosis in HUVECs

**Figure 3:**
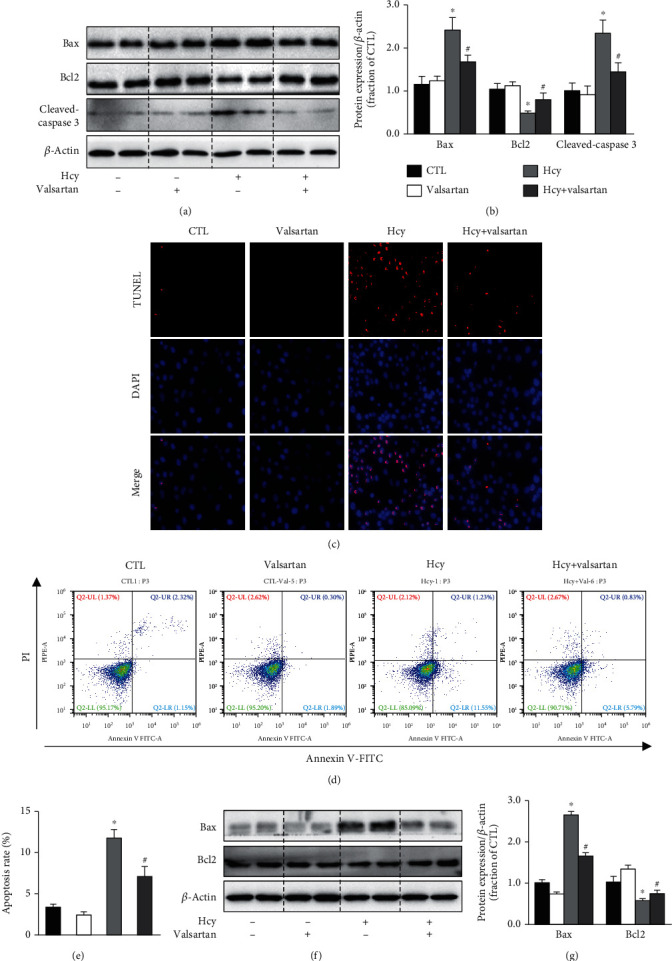
Valsartan prevented Hcy-induced apoptosis in HUVECs. (a, b) Representative immunoblots and corresponding densitometry analysis of Bax, Bcl2, and cleaved-caspase 3 protein in Hcy-treated HUVECs with or without valsartan. (c) Representative photomicrographs of TUNEL staining the HUVECs in CTL, valsartan, Hcy, and Hcy+valsartan groups. (d, e) Apoptosis was detected by flow cytometry using Annexin V-FITC/PI apoptosis detection kit in Hcy-treated HUVECs with valsartan. (f, g) Representative immunoblots and corresponding densitometry analysis of Bax, Bcl2, and cleaved-caspase 3 protein in Hcy pretreated HUVECs with or without valsartan. Data are shown as mean ± SEM; ^∗^*P* < 0.05 compared with the CTL group; ^#^*P* < 0.05 compared with the Hcy group.

To determine the role of AT1 receptor in Hcy-induced HUVEC injury, apoptosis-related proteins were next investigated. The protein abundance of Bax was markedly increased after Hcy treatment, whereas Bcl2 protein expression was significantly decreased, both of which were obviously prevented by valsartan treatment (Figures 3(a) and 3(b)). Valsartan was also shown to attenuate protein expression of cleaved-caspase 3 induced by Hcy, indicating amelioration of HUVEC injury. TUNEL assay showed marked cell death in HUVEC cells treated with Hcy, which were obviously decreased by valsartan treatment (Figure 3(c)). Annexin V-FITC/PI double staining also showed that valsartan at least partially prevented Hcy-induced cell apoptosis in HUVECs (Figures 3(d) and 3(e)). In the condition that HUVECs were preinjured by Hcy, valsartan still showed protection, as increased Bax and decreased Bcl2 protein expression after Hcy was markedly prevented (Figures 3(f) and 3(g)) (protocol 2). These findings strongly suggest a protective role of AT1 receptor blockade in HUVEC injury induced by Hcy.

### 3.4. Knockdown of AT1 Receptor Prevented Hcy-Induced Impaired Autophagy, ER Stress, and Apoptosis in HUVECs

**Figure 4:**
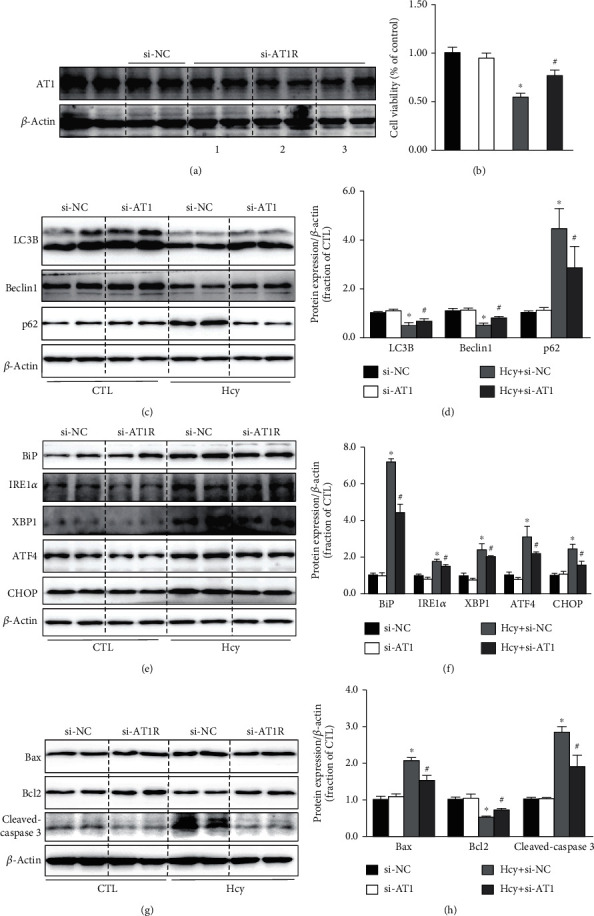
Knockdown of AT1 receptor prevented Hcy-induced impaired autophagy, ER stress, and apoptosis in HUVECs. (a) Representative immunoblots analysis of AT1 protein in HUVECs transfected with AT1 siRNA. (b) Cell viability was assessed by CCK8 assay in Hcy-treat HUVEC cells with AT1 knockdown. (c, d) Representative immunoblots and corresponding densitometry analysis of LC3B, Beclin1, and p62 protein in Hcy-treated HUVECs with or without AT1-siRNA. (e, f) Representative immunoblots and corresponding densitometry analysis of BiP, IRE1*α*, XBP1, ATF4, and CHOP protein in Hcy-treated HUVECs with or without AT1-siRNA. (g, h) Representative immunoblots and corresponding densitometry analysis of Bax, Bcl2, and cleaved-caspase 3 protein in Hcy-treated HUVECs with or without AT1-siRNA. Data are shown as mean ± SEM; ^∗^*P* < 0.05 compared with si-NC group; ^#^*P* < 0.05 compared with Hcy+si-NC group.

To further verify the role of AT1 receptors in Hcy-induced impaired autophagy, ER stress, and apoptosis in HUVECs, AT1 receptor was knocked down in HUVECs by small interfering RNAs (siRNAs) targeting AT1 receptor. siRNA-AT1-2 showed the most significant inhibition to AT1R expression, which was used in subsequent experiments (Figure 4(a)). Cell viability assay showed that inhibition of AT1 receptor by siRNA did not affect HUVEC survival, but it prevented cell injury induced by Hcy (Figure 4(b)). AT1 knockdown markedly improved Hcy-induced impaired autophagy (Figures 4(c) and 4(d)), ER stress (Figures 4(e) and 4(f)), and apoptosis (Figures 4(g) and 4(h)). Taken together, these data suggest that activation of AT1 receptor may be involved in the modulation of Hcy-induced impaired autophagy, ER stress, and apoptosis in HUVECs.

### 3.5. AT1-GSK3*β*-mTOR Signaling Was Involved in Hcy-Induced HUVEC Injury

**Figure 5:**
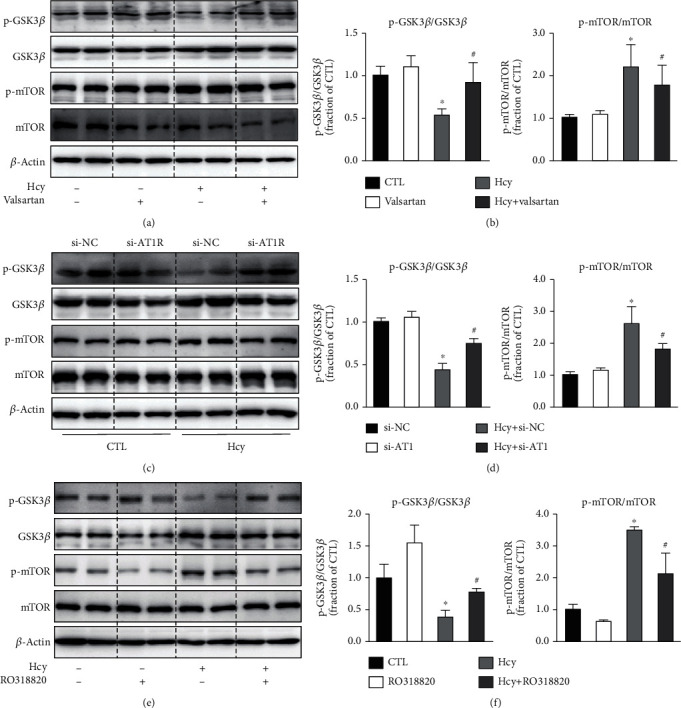
AT1 receptor-GSK3*β*-mTOR signaling was involved in Hcy-induced HUVEC injury. (a, b) Representative immunoblots and densitometry ratio of p-GSK3*β*/GSK3*β* and p-mTOR/mTOR in Hcy-treated HUVECs pretreated with or without valsartan. (c, d) Representative immunoblots and densitometry ratio of p-GSK3*β*/GSK3*β* and p-mTOR/mTOR in Hcy-treated HUVECs with or without AT1-siRNA. (e, f) Representative immunoblots and densitometry ratio of p-GSK3*β*/GSK3*β* and p-mTOR/mTOR in Hcy-treated HUVECs with or without RO318820. Data are shown as mean ± SEM; ^∗^*P* < 0.05 compared with CTL group; ^#^*P* < 0.05 compared with Hcy group; ^∗^*P* < 0.05 compared with si-NC group; ^#^*P* < 0.05 compared with Hcy+si-NC group.

**Figure 6:**
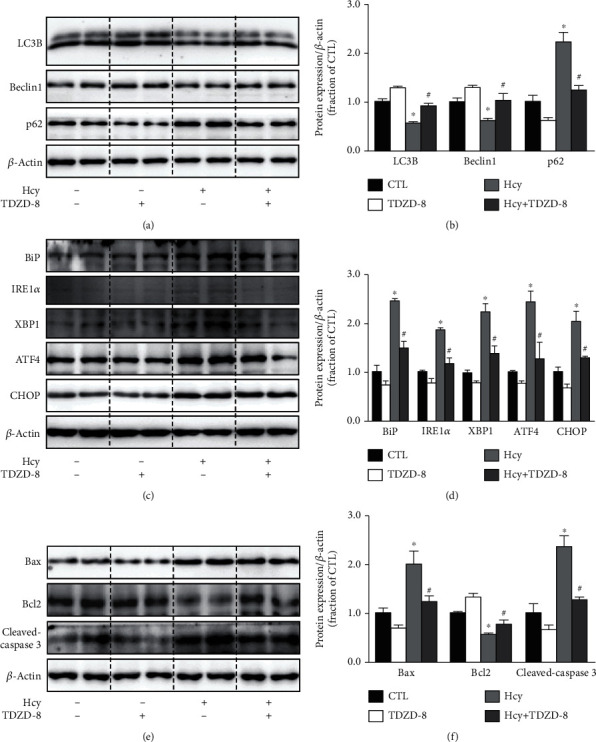
GSK-3*β* inhibitor TDZD-8 treatment reversed Hcy-induced impaired autophagy, ER stress, and apoptosis in HUVECs. (a, b) Representative immunoblots and corresponding densitometry analysis of LC3B, Beclin1, and p62 protein in Hcy-treated HUVECs with or without TDZD-8. (c, d) Representative immunoblots and corresponding densitometry analysis of BiP, IRE1*α*, XBP1, ATF4, and CHOP protein in Hcy-treated HUVECs with or without TDZD-8. (e, f) Representative immunoblots and corresponding densitometry analysis of Bax, Bcl2, and cleaved-caspase 3 protein in Hcy-treated HUVECs with or without TDZD-8. Data are shown as mean ± SEM; ^∗^*P* < 0.05 compared with the CTL group; ^#^*P* < 0.05 compared with the Hcy group.

Next, we examined the downstream signaling pathway that probably mediates AT1 activation-induced HUVEC injury. GSK-3*β* inhibits autophagy through suppressing the mammalian target of rapamycin (mTOR) complex 1 [20]. Our previous study demonstrated that GSK-3*β* inhibition enhanced autophagy and improved cell survival in acute kidney injury [21]. The abundance of phosphorylated GSK3*β* at Ser9 was markedly decreased, while the total GSK3*β* protein level was slightly increased after Hcy treatment in HUVECs. Valsartan markedly prevented decreased ratio of p-GSK3*β* to GSK3*β* (Figures 5(a) and 5(b)). Similarly, the protein abundance of phosphorylated mTOR was also increased after Hcy treatment in HUVECs, indicating an activation of mTOR signaling induced by Hcy. Blockade of AT1 by valsartan significantly decreased the protein expression of p-mTOR (Figures 5(a) and 5(b)). As expected, AT1 knockdown prevented Hcy-induced decreased ratio of p-GSK3*β* to GSK3*β* and decreased expression of p-mTOR (Figures 5(c) and 5(d)) (protocol 1). As a downstream signaling of a G-protein coupled receptor, PKC pathway is well known to mediate the biological effects of angiotensin-AT1R. Interestingly, an inhibitor of PKC RO318820 was shown to significantly increase the ratio of p-GSK3*β*/GSK3*β* and decreased mTOR protein expression after Hcy treatment (Figures 5(e) and 5(f)) (protocol 3), likely indicating an important role of AT1-PKC pathway in Hcy-induced activation of GSK3*β* signaling. Furthermore, GSK3*β* inhibitor TDZD-8 treatment reversed Hcy-induced induction of impaired autophagy (Figures 6(a) and 6(b)), ER stress (Figures 6(c) and 6(d)) (protocol 3), and apoptosis (Figures 6(e) and 6(f)), suggesting that inhibition of GSK3*β* prevented Hcy-induced HUVEC injuries.

### 3.6. Autophagy Inhibition by 3-MA or Inhibition of ER Stress by 4-PBA Prevented Hcy-Induced HUVEC Injury

**Figure 7:**
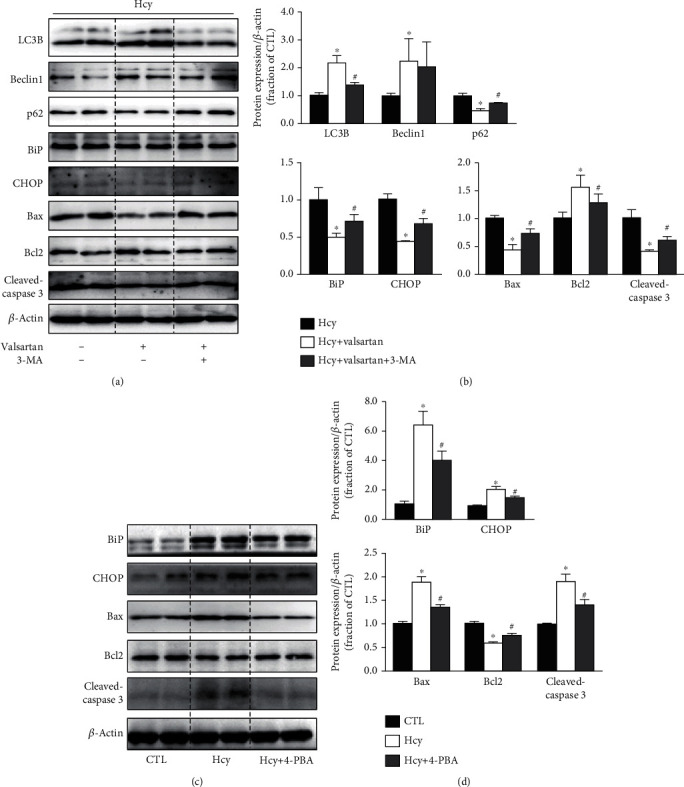
Autophagy inhibition by 3-MA and inhibition of ER stress by 4-PBA inhibited ER stress in HUVECs. (a, b) Representative immunoblots and corresponding densitometry analysis of LC3B, Beclin1, p62, BiP, CHOP, Bax, Bcl2, and cleaved-caspase 3 protein in Hcy-treated HUVECs with or without valsartan or 3-MA. (c, d) Representative immunoblots and corresponding densitometry analysis of BiP, CHOP, Bax, Bcl2, and cleaved-caspase 3 protein in Hcy-treated HUVECs with or without 4-PBA. Data are shown as mean ± SEM; ^∗^*P* < 0.05 compared with the CTL group; ^#^*P* < 0.05 compared with the Hcy group.

Next, 3-methyladenine (3-MA), an inhibitor of autophagosome formation, was used to further verify the role of autophagy in HUVEC injured by Hcy. Valsartan induced upregulation of LC3B and Beclin1 and downregulation of p62 in Hcy-treated HUVECs; 3-MA markedly inhibited these changes (Figures 7(a) and 7(b)). 3-MA also markedly exacerbated ER stress and apoptosis (Figures 7(a) and 7(b)) compared with Hcy+Valsartan group (protocol 4). These findings potentially suggested that inhibition of autophagy by 3-MA reversed the protection of valsartan in HUVECs treated with Hcy. The above data support the conception that impaired autophagy may induce ER stress, leading to apoptosis in HUVECs treated with Hcy; would inhibition of ER stress decrease apoptosis in Hcy groups? 4-PBA, a chaperon to attenuate ER stress, significantly decreased protein abundance of BiP and CHOP in HUVECs treated with Hcy (Figures 7(c) and 7(d)). 4-PBA could further decrease protein expression of Bax and cleaved-caspase 3 while increasing the abundances of Bcl2 protein in HUVECs treated with Hcy (Figures 7(c) and 7(d)) (protocol 4). These findings indicated that rescue of ER stress may prevent HUVEC cell apoptosis induced by Hcy.

## 4. Discussion

Homocysteine is a sulfur-containing amino acid formed during the metabolism of methionine to cysteine. Hcy is considered as an independent risk factor for atherosclerosis and cardiovascular diseases and Hcy exerts its adverse effects by disturbing endothelial function [22, 23]. Endothelial cell apoptosis is a hallmark of atherosclerotic lesions [24]. Early studies showed that Hcy induced ER stress in HUVEC [25, 26], which leads to apoptosis [23] in vascular endothelia and contributes to the development of atherosclerosis. In the current study, we confirmed that Hcy caused ER stress (as seen in increased protein expression of ER stress markers) in a time-dependent pattern, which was associated with apoptosis in HUVECs. Interestingly, 4-PBA, a molecular chaperone-like fatty acid, effectively attenuated ER stress and apoptosis in HUVECs, supporting a role of ER stress in cell injury induced by Hcy.

We further demonstrated that impaired autophagy may be a potential mechanism that caused ER stress induced by Hcy in HUVECs. After Hcy treatment, protein expression of LC3B and Beclin1 was significantly decreased, and the numbers of LC3B puncta were reduced, indicating an inhibition of autophagy or impaired autophagy in HUVECs. Autophagy, primarily, acts as a survival mechanism under conditions of stress, maintaining cellular homeostasis by regenerating metabolic precursors and clearing subcellular debris [27], which helps to relieve the stress in the cell and reinstate homeostasis. Impaired autophagy is supposed to compromise cellular homeostasis and lead to cell injury or death. ER stress and autophagy are individually complex systems and function independently, but they interact with each other in many conditions. A large number of evidences have shown that autophagy is activated in response to ER stress. [11, 28] However, we recently demonstrated that impaired autophagy induced by palmitic acid actually caused ER stress and apoptosis [10]. Impaired autophagy or autophagic flux stagnation induced by stress (e.g., lipid) may result in the accumulation of abnormal proteins [29]. It is reasonable to assume that unstable proteostasis and insufficient autophagy after Hcy treatment may increase the burden of ER, causing ER stress and apoptosis in HUVEC. This was further supported by the finding that inhibition of autophagy with 3-MA aggravated ER stress and apoptosis induced by Hcy.

**Figure 8:**
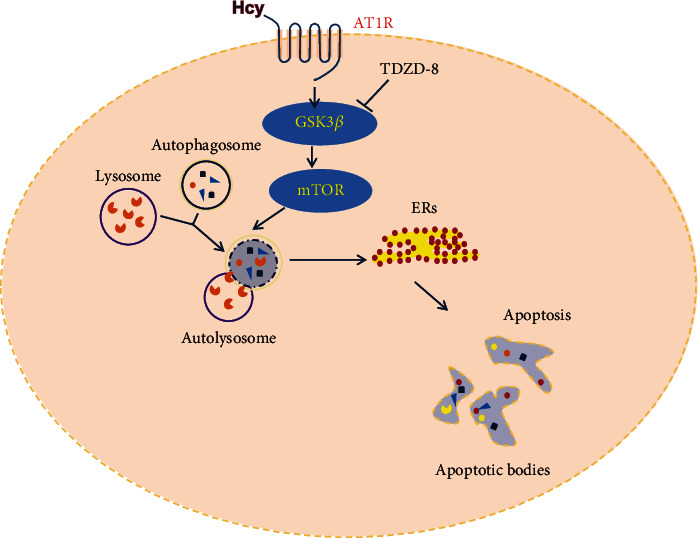
A potential mechanism by which Hcy caused injury in HUVECs. Hcy-induced impaired autophagy and ER stress in HUVECs, which was associated with increased protein expression levels of AT1. AT1 block or silence improved autophagy and ER stress and attenuated apoptosis, at least partially, through suppressing GSK-3*β*/mTOR signaling pathway in HUVEC cells. Hcy: homocysteine; AT1: angiotensin II type 1 receptor; TDZD-8: 4-benzyl-2-methyl-1,2,4-thiadiazolidine-3,5-dione.

How does Hcy inhibit autophagy in endothelial cells? mTOR is an evolutionarily conserved serine/threonine protein kinase that regulates multiple cellular processes including autophagy. Autophagy is initiated by the dephosphorylation of mTOR and induction of ATG1/ULK complex [20]. As such, mTOR suppression by some chemicals (e.g., rapamycin) stimulates autophagy. Hcy has been found to inhibit autophagy through mTOR in neurons [30] and aortic valve interstitial cells [31]. GSK-3*β* is involved in the regulation of autophagy. Overexpression of GSK-3*β* activates mTOR complex 1 and suppresses autophagy, while inhibition of GSK-3*β* with inhibitors increases autophagic flux [32]. Our recent study demonstrated that inhibition of GSK-3*β* activated autophagy and suppressed the release of inflammatory factors [21]. These studies highlight an important role of GSK-3*β*-mTOR pathway in autophagy modulation. In HUVECs treated with Hcy, p-GSK3*β*/GSK3*β* was significantly decreased, which was associated with increased p-mTOR/mTOR. To support this, a GSK inhibitor TDZD showed a protection in reversing Hcy-induced impaired autophagy and ER stress. Therefore, Hcy likely through GSK-3*β*-mTOR signaling pathway suppresses autophagy in HUVECs (Figure 8).

The other remarkable finding in the current study is local RAS activation in HUVECs after Hcy treatment. Clinical and experimental evidence indicates that Hcy and activation of the renin-angiotensin system, mediated by AT1 receptor, are involved in a variety of vascular pathologies [14]. Previous studies demonstrated that homocysteine directly interacted with and activated the AT1 receptor to aggravate vascular injury. Hcy was shown to be an endogenous ligand and partial agonist of the AT1 receptor. Binding to AT1 receptor, Hcy causes a distinct conformational change of the AT1 receptor [18]. In the current study, Hcy notably increased the protein expression levels of AT1, which was associated with impaired autophagy and ER stress in HUVECs. When blocking AT1 with valsartan or silencing AT1 with siRNA, autophagy was improved, and ER stress was relieved, followed by attenuated apoptosis (Figure 8). These data support a role of AT1R in mediating Hcy-induced injury. PKC signaling has been shown activated by Hcy in vascular injury, although not as predominant as angiotensin II [18]. Interestingly, in the present study, Hcy-induced HUVEC injury was likely through activating PKC pathway, a downstream signaling of AT1R, as PKC inhibition improved autophagy and ER stress; however, the detailed mechanism behind this was not examined.

Together with other studies [14, 18, 33], our data may have implications in treating vascular injuries in patients with hyperhomocysteinemia (HHcy) and hypertensive people with HHcy. The interaction between Hcy and AT1R activation provides an option that blockade of RAS, in particular, blocking AT1R (e.g., sartan family), may be considered as an intervention strategy for these patients, in addition to lowering total plasma Hcy. Whether Hcy induced angiotensin II production was not examined in the present study; however, enalapril was shown not to inhibit Hcy-induced AT1 receptor activation and HHcy-induced vascular injury [18], indicating that blockade of AT1R with ARB may be a better choice. More evidence is warranted apparently. In addition, several other potential strategies are also indicated in our study. As impaired autophagy and unresolved ER stress were seen in Hcy-induced cell injury, it is reasonable to target these cellular events, for example, using molecular chaperons to attenuate ER stress may prevent further damage of endothelia induced by Hcy. Adequate induction of autophagy might be protective in Hcy-induced injury, but targeting autophagy as a therapeutic intervention may be challenging. The outcome of nonspecific activation of autophagy will need to be carefully evaluated, as autophagy is generally considered as a “double-edged sword”.

In conclusion, Hcy treatment induced impaired autophagy, ER stress, and apoptosis in HUVECs, which was likely attributed to the activation of the AT1 receptor, at least partially, through GSK-3*β*/mTOR signaling pathway (Figure 8). Despite AT1R blocker, targeting to rescue autophagy or ER stress may also be therapeutic options in patients with HHcy, and more vigorous studies are absolutely needed though.

## Data Availability

The analyzed data sets generated during the study are available from the corresponding author on reasonable request.
